# Supplementary material for: Analysis of PPARγ Signaling Activity in Psoriasis
Source: Int J Mol Sci. 2021 Aug 10;22(16):8603. doi: 10.3390/ijms22168603 (PMC8395241; doi:10.3390/ijms22168603)
Supplement: Supplementary file 1 [file ijms-22-08603-s001.zip › Supplemental materials_Analysis of PPARg signaling activity in psoriasis/Pathway models/Models images and html files/Interleukin-17 and interleukin-22 signaling in psoriasis/9343.html]

MAP2K7


# Protein MAP2K7

|  |  |
| --- | --- |
| URN | urn:agi-llid:5609 |
| Total Entities | 0 |
| Connectivity | 846 |
| Name | MAP2K7 |
| Description | mitogen-activated protein kinase kinase 7 |
| Notes | The protein encoded by this gene is a dual specificity protein kinase that belongs to the MAP kinase kinase family. This kinase specifically activates MAPK8/JNK1 and MAPK9/JNK2, and this kinase itself is phosphorylated and activated by MAP kinase kinase kinases including MAP3K1/MEKK1, MAP3K2/MEKK2,MAP3K3/MEKK5, and MAP4K2/GCK. This kinase is involved in the signal transduction mediating the cell responses to proinflammatory cytokines, and environmental stresses. Alternative splicing results in multiple transcript variants. [provided by RefSeq, Jul 2014] |
| Primary Cell Localization | Cytoplasm |
| Class | Protein kinase |

---

|  |  |
| --- | --- |
| Pathway | Osteoarthritis |
|  | Skin Aging |
|  | Thrombopoietin Receptors Signaling in Platelet Maturation |
|  | Toll-like Receptors Act through MYD88-TIRAP Signaling |
|  | TLR4 Signaling in Leukocytes |
|  | T-Cell Dependent B-Cell Activation |
|  | T-Cell Independent B-Cell Activation |
|  | Peripheral T-Cell Tolerance |
|  | Th1-Cell Differentiation |
|  | Treg-Cell Differentiation |
|  | Th2-Cell Differentiation |
|  | T-Cell Receptor Signaling |
|  | Plasmin Effects in Inflammation |
|  | MC1R in anti-Inflammatory Signaling |
|  | Basophil Activation |
|  | Mast-Cell Activation via IgE Signaling |
|  | Mast-Cells De Novo Synthesized Mediators via IgE Independent Signaling |
|  | Neutrophil Recruitment and Priming |
|  | Nociception Expression Targets Signaling |
|  | ADRA1A -> IL6 Production |
|  | CNR1/2 -> IL1B/2/4/6/10 Production |
|  | DRD2 Expression Targets |
|  | PTGER2/3 -> Inflammation-Related Expression Targets |
|  | Circadian Clock Genes in Suprachiasmatic Nuclei Neurons |
|  | Apoptosis |
|  | Th1-Cells Activation and Proliferation in Atherosclerosis |
|  | Inflammatory Reaction in Acne Vulgaris |
|  | Atopic Dermatitis Onset |
|  | Mast-Cells Activation in Atopic Dermatitis |
|  | IL17 Signaling in Psoriasis |
|  | Th0-Cell Aberrant Activation in Systemic Lupus Erythematosus |
|  | B-Cell Activation in Systemic Lupus Erythematosus |
|  | Th2-Cell Function in Systemic Lupus Erythematosus |
|  | Acute Cytotoxic CD8+T-Cell Response against Melanocytes |
|  | Vitiligo |
|  | T-Cell Cytotoxic Response against Melanocytes in Vitiligo |
|  | Apoptosis of Melanocyte in Vitiligo |
|  | Humoral Immunity in Vitiligo |
|  | Treg-Cell Function in Diabetes Mellitus Type 1 |
|  | Natural Killer T-Cell Roles in Diabetes Mellitus Type 1 |
|  | non-Suppressive Treg-Cell in Diabetes Mellitus Type 1 |
|  | TLR2 Signaling in Treg-Cell in Type 1 Diabetes (Animal Model) |
|  | Treg-Cell Activation in Diabetes Mellitus |
|  | beta-Cell Destruction through Cytokines in Diabetes Mellitus |
|  | Apoptosis of Thyroid Cell in Hashimoto's Thyroiditis |
|  | GNRH Signaling Impairment in Hypogonadotrophic Hypogonadism |
|  | CD4+T-Cell Response in Celiac Disease |
|  | OipA Signaling in Helicobacter Pylori Infection |
|  | Th2-Cell Function in Ulcerative Colitis |
|  | T-Cell Cytotoxic Response in Ulcerative Colitis |
|  | Osteoclast Activation in Multiple Myeloma |
|  | SERPINA1 Associated Liver Damage |
|  | Pancreatic Ductal Carcinoma |
|  | Pancreatic Neuroendocrine Tumors |
|  | TGFBR Signaling in Pancreatic Neoplasms |
|  | IgE Induces Airway Smooth Muscle Cell Proliferation |
|  | Basophil Activation in Asthma |
|  | Mast-Cell Activation in Asthma |
|  | Th2-Cell Response in Asthma |
|  | Mucin Production in Goblet Airway Epithelial Cells |
|  | Osteoclast Activation in Gout |
|  | TNF and IL1B Induce Metalloproteinase Synthesis in Osteoarthritis |
|  | Apoptosis of Chondrocyte |
|  | Dendritic Cell Dysfunction in Psoriatic Arthritis |
|  | Synovial Fibroblast Activation in Psoriatic Arthritis |
|  | Keratinocyte Activation in Psoriatic Arthritis |
|  | PTPN22 Role in Rheumatoid Arthritis |
|  | Osteoclast Activation in Rheumatoid Arthritis |
|  | Synovial Fibroblast Activation by Citokines in Rheumatoid Arthritis |
|  | Apoptosis Block in Synovial Fibroblast |
|  | FAS Mediated Apoptosis in Systemic Scleroderma |
|  | B-Cells Function in Systemic Scleroderma |
|  | Th2-Cells Function in Systemic Scleroderma |
|  | Skin Fibrosis |
|  | CCR5 Signaling in Macrophages |
|  | CD4+ T-Cell Function Decline in HIV |
|  | Apoptosis Block in Infected Cells |
|  | CD4+ T-Cell Death in HIV |
|  | TNF Receptor Associated Periodic Syndrome |
|  | ER Stress in Huntington Disease |
|  | Osteoclast Activation in Postmenopause |
|  | Glioma Invasion Signaling |
|  | Growth Factor Signaling in Neuroblastoma |
|  | Neuroblastoma |
|  | WNT Signaling in Breast Cancer |
|  | Angiogenesis in Endometriosis |
|  | Subepithelial Fibroblasts in Middle Ear Cholesteatoma |
|  | HPV E5 Regulates EDNRA and EGFR Signaling Pathway in Keratinocytes |
|  | ER Stress in Macular Degeneration |
|  | MYOC Associated Glaucoma |
|  | Immune System Role in Otitis Media |
|  | Alveolar Macrophage Negative Regulation Declined in COPD |
|  | Alveolar Macrophages Dysfunction in COPD |
|  | T-Cell Cytotoxic Activation |
|  | Mucin Hyperproduction in Goblet and Mucous Cells |
|  | WNT Signaling in Cystic Kidney Disease |
|  | Plasmin Role in Glomerulonephritis |
|  | Podocyte Dysfunction in Glomerulonephritis |
|  | CD38/CD19 -> JUN/FOS/NF-kB Signaling in B-cell Proliferation |
|  | ANGPT2 -> AP-1/CREBBP/MYC Expression Targets |
|  | AREG -> AP-1 Expression Targets |
|  | Dopamine/Gi Expression Targets |
|  | BTC -> AP-1/ATF/CREB Expression Targets |
|  | Frizzled Receptors -> ARRB1/ARRB2 non-Canonical Signaling |
|  | Complement Component GPCRs Signaling |
|  | EGF -> AP-1/ATF Expression Targets |
|  | EGF -> CREB/CREBBP/ELK/SRF/MYC Expression Targets |
|  | EGF -> TP53 Expression Targets |
|  | EGFR/ERBB2 -> TP53 Signaling |
|  | EGFR -> AP-1/ATF2 Signaling |
|  | EGFR -> AP-1/CREB/ELK/SRF/MYC Signaling |
|  | EREG -> AP-1/ATF Expression Targets |
|  | Erythropoietin -> AP-1/MYC/CREB Expression Targets |
|  | F2 -> AP-1/CREB/ELK/SRF/SP1 Expression Targets |
|  | PLG -> AP-1/CREB/ELK/SRF/SP1 Expression Targets |
|  | FGF9 -> AP-1/CREB/MYC Expression Targets |
|  | FGF10 -> AP-1/CREB/CREBBP/MYC Expression Targets |
|  | FGF1 -> AP-1/CREB/ELK/SRF/MYC Expression Targets |
|  | FGF8 -> AP-1/CREB/MYC Expression Targets |
|  | FGF18 -> AP-1/CREB Expression Targets |
|  | FGF4 -> AP-1/MYC Expression Targets |
|  | FGF7 -> AP-1/CREB/CREBBP/MYC Expression Targets |
|  | FGF2 -> AP-1/CREB/CREBBP/ELK/SRF/MYC Expression Targets |
|  | FGFR -> AP-1/CREB/CREBBP/ELK/SRF/MYC Signaling |
|  | FrizzledR -> JUN/PAX2 Signaling |
|  | HBEGF -> AP-1/ATF Expression Targets |
|  | HBEGF -> TP53 Expression Targets |
|  | HBEGF -> CREB/MYC Expression Targets |
|  | HGF -> AP-1/CREB/ELK/SRF/MYC Expression Targets |
|  | HGFR -> AP-1/CREB/MYC Signaling |
|  | RAS/RAF/MAPK Signaling Activation in Cancer |
|  | RAS/RAF/MAPK Signaling Activation by Blocking of Tumor Suppressors |
|  | Apoptosis Block by Death Receptor Signaling in Cancer |
|  | Apoptosis Evasion in Cancer: Overview |
|  | VEGFA Dependent Angiogenesis in Cancer |
|  | VEGF Independent Angiogenesis in Cancer |
|  | Effector T-cell Inactivation in Cancer Immune Escape |
|  | FibronectinR -> AP-1/ELK/SRF/SREBF Signaling |
|  | CTGF -> AP-1/CREB/MYC Expression Targets |
|  | PAF/Gq -> AP-1/ATF1/CREB/ERK/SRF Expression Targets |
|  | Dronabinol/Anandamide Expression Targets |
|  | S1P Expression Targets |
|  | NRG1 -> CREB/CREBBP/ELK/SRF/MYC Expression Targets |
|  | NRG1 -> TP53 Expression Target |
|  | NRG1 -> AP-1/ATF Expression Targets |
|  | NGF -> SMAD3/NF-kB Expression Targets |
|  | NGF -> AP-1/TP53/MYC Expression Targets |
|  | BDNF Expression Targets |
|  | NTF3 Expression Targets |
|  | NTF4 Expression Targets |
|  | NOTCH -> TCF3 Signaling |
|  | NOTCH Expression Targets |
|  | PDGFB -> AP-1/CREB/MYC Expression Target |
|  | PDGFD -> AP-1 Expression Targets |
|  | PDGF -> AP-1/CREB/CREBBP/MYC Expression Targets |
|  | PDGFR -> AP-1/MYC Signaling |
|  | AVP/Gs -> CREB/ELK/SRF/AP-1/EGR Expression Targets |
|  | GNRH1 Expression Targets |
|  | GNRH2 Expression Targets |
|  | EDN1 Expression Targets |
|  | EDN3 Expression Targets |
|  | AVP/Gq -> CREB/ELK/SRF/AP-1/EGR Expression Targets |
|  | AngiopoietinR -> AP-1 Signaling |
|  | GDNF -> HSF1 Signaling |
|  | EphrinB -> MAPK/JUN/FOS Signaling |
|  | NTRK -> AP-1/CREB/ELK/SRF/MYC/SMAD3/TP53 Signaling |
|  | GAS6 -> AP-1/CREB Expression Targets |
|  | FIGF -> AP-1 Expression Target |
|  | KITLG -> AP-1/CREB/CREBBP/MYC Expression Targets |
|  | GDNF -> HSF1 Expression Targets |
|  | CSF1 -> AP-1/CREB/CREBBP/MYC Expression Targets |
|  | PGF -> AP-1/CREB/CREBBP/MYC Expression Targets |
|  | FLT3LG -> AP-1/CREB/CREBBP Expression Targets |
|  | TNF-alpha/TNFRSF1A Signaling |
|  | JNK/MAPK Signaling |
|  | VEGF Signaling |
|  | EGFR Signaling |
|  | WNT Planar Cell Polarity (PCP) non-Canonical Signaling |
|  | T-Cell Receptor -> AP-1 Signaling |
|  | TGFA -> TP53 Expression Targets |
|  | TGFA/AP-1/ATF Expression Targets |
|  | TGFA -> CREB/CREBBP/ELK-SRF/MYC Expression Targets |
|  | FASLG Expression Targets |
|  | TNFSF10 Expression Targets |
|  | LTA Expression Targets |
|  | TNFSF14 Expression Targets |
|  | TNF -> AP-1 Expression Targets |
|  | CD72 -> AP-1 Expression Targets |
|  | CD40LG -> NF-kB/ELK/SRF -> CREB/NFATC Expression Targets |
|  | TNFSF13 Expression Targets |
|  | TNFSF13B Expression Targets |
|  | TNF -> ELK-SRF Expression Targets |
|  | TNF -> TP53/ATF Expression Targets |
|  | EDA Expression Targets |
|  | EctodysplasinR -> AP-1 Signaling |
|  | TNFR -> CREB/ELK-SRF Signaling |
|  | TNFR -> AP-1/ATF/TP53 Signaling |
|  | NGFR -> AP-1/CEBPB/CREB/ELK/SRF/TP53 Signaling |
|  | TNFRSF6 -> DDIT3 Signaling |
|  | TNFRSF6 -> HSF1 Signaling |
|  | TNFRSF1A -> AP-1/ATF/TP53 Signaling |
|  | TNFRSF1A -> CREB/ELK-SRF Signaling |
|  | Thrombopoietin -> SPI1 Expression Targets |
|  | Thrombopoietin -> AP-1/CREB/CREBBP/MYC Expression Targets |
|  | IL1B -> PGE2 Expression Targets |
|  | ROS in Angiotensin Mediated Cardiovascular Remodeling and Hypertrophy |
|  | VEGFA -> AP-1/CREBBP/MYC Expression Targets |
|  | VEGFR -> AP-1/CREB/MYC Signaling |
|  | WNT1 Expression Targets |
|  | WNT3A Expression Targets |
|  | WNT4 Expression Targets |
|  | WNT5A Expression Targets |
|  | WNT7A Expression Targets |
|  | 1\_Inflammation in keratinocytes in the pathology of acne |
|  | 2\_Acute phase initiation in atopic dermatitis |
|  | 2\_Interleukin-17 and interleukin-22 signaling in psoriasis |
|  | 1\_The onset of atopic dermatitis |
|  | 1\_TNF and IL1B provoke ECM degradation in osteoarthritis |
|  | 1\_Osteoclast-mediated bone resorption in postmenopausal women |
|  | 2\_Chondrocyte death in osteoarthritis |
|  | 1\_Formation of cysts in polycystic kidney disease |
|  | 3\_Mesangial cell dysfunction in glomerulonephritis |
|  | 2\_Podocyte dysfunction in glomerulonephritis |
|  | 1\_Disruption of luteinizing hormone and follicle-stimulating hormone secretion in PCOS |
|  | 2\_MYOC-associated glaucoma |
|  | 3\_Endoplasmic reticulum stress in age-related macular degeneration |
|  | 2\_Cigarette smoke and inflammation cause alveolar epithelial cell death in COPD |
|  | 3\_Role of alveolar macrophages in pulmonary tissue destruction in COPD |
|  | 2\_Lipotoxicity in NAFLD |
|  | 2\_HIV - induced depletion of CD4+ T-cells |
|  | 4\_3\_Insulin resistance: FFA-related insulin resistance |
|  | 2\_SERPINA1-associated liver damage |
|  | mapk1 protein modificators |
|  | Interleukin-17 and interleukin-22 signaling in psoriasis |

---

|  |  |
| --- | --- |
| MedScan ID | 5609 |

---

|  |  |
| --- | --- |
| LocusLink ID | 5609 |
|  | 26400 |
|  | 363855 |

---

|  |  |
| --- | --- |
| Alias | JNKK2 |
|  | MAPK kinase 7 |
|  | Dual specificity mitogen-activated protein kinase kinase 7 |
|  | 5930412N11 |
|  | c-Jun N-terminal kinase kinase II |
|  | JNK kinase 2 |
|  | stress-activated protein kinase kinase-4 SKK4 |
|  | ERK kinase VII |
|  | MAP kinase kinase VII |
|  | MAPK/ERK kinase VII |
|  | Map2k7 |
|  | JNK kinase II |
|  | mitogen-activated protein kinase kinase VII |
|  | OTTMUSP00000045502 |
|  | OTTMUSP00000017733 |
|  | MEK 7 |
|  | OTTMUSP00000022436 |
|  | OTTMUSP00000017734 |
|  | protein kinase, mitogen-activated, kinase VII |
|  | stress-activated protein kinase kinase 4 |
|  | c-Jun N-terminal kinase kinase 2 |
|  | MAP kinase kinase 7 |
|  | PRKMK7 |
|  | MAPK/ERK kinase 7 |
|  | MKK7/JNKK2/SKK4 |
|  | MKK7 |
|  | LOC199677 |
|  | mitogen activated protein kinase kinase 7 |
|  | SAPKK4 |
|  | protein kinase, mitogen activated, kinase 7 |
|  | MAPK kinase VII |
|  | JNK activating kinase II |
|  | Dual specificity mitogen-activated protein kinase kinase VII |
|  | 5930412N11Rik |
|  | OTTMUSP00000017732 |
|  | OTTMUSP00000022434 |
|  | OTTHUMP00000174397 |
|  | JNK activating kinase 2 |
|  | ERK kinase 7 |
|  | Sek2 |
|  | SKK4/MKK7 |
|  | MKK7a |
|  | OTTMUSP00000022437 |
|  | MAPKK7 |
|  | OTTMUSP00000022435 |
|  | SAPK kinase 4 |
|  | MEK |
|  | SAPKK-4 |
|  | JNK-activating kinase 2 |
|  | Mek7 |
|  | JNKK 2 |
|  | MAPKK 7 |
|  | JNKK |
|  | SKK4 |
|  | 2 |
|  | map2k7 {ECO:0000312\|EMBL:AAX61178.1} |
|  | mitogen-activated protein kinase kinase 7 |
|  | JNK activating kinase 2 |
|  | JNK kinase II |
|  | JNK activating kinase II |
|  | JNK kinase 2 |
|  | protein kinase, mitogen-activated, kinase 7 |
|  | MAPKK7s |
|  | PRKMK7s |

---

|  |  |
| --- | --- |
| EC Number | 2.7.12.2 |

---

|  |  |
| --- | --- |
| GO ID | 0005524 |
|  | 0008545 |
|  | 0004708 |
|  | 0019899 |
|  | 0000287 |
|  | 0019901 |
|  | 0019903 |
|  | 0004674 |
|  | 0004713 |
|  | 0038095 |
|  | 0007254 |
|  | 0007257 |
|  | 0000187 |
|  | 0032147 |
|  | 0006915 |
|  | 0018108 |
|  | 0070374 |
|  | 0051973 |
|  | 1904355 |
|  | 0032212 |
|  | 0045893 |
|  | 0009411 |
|  | 0009408 |
|  | 0006970 |
|  | 0034612 |
|  | 0007165 |
|  | 0023014 |
|  | 0051403 |
|  | 0031098 |
|  | 0005737 |
|  | 0005829 |
|  | 0005634 |
|  | 0031435 |
|  | 0008022 |
|  | 0043525 |
|  | 0001934 |
|  | 0006468 |
|  | 2000671 |
|  | 0009611 |
|  | 0072709 |
|  | 0004702 |
|  | 0035897 |
|  | 0016301 |
|  | 0046872 |
|  | 0000166 |
|  | 0004672 |
|  | 0016740 |
|  | 0016310 |
|  | 0006950 |
|  | 0007346 |
|  | 0005622 |
|  | 0002755 |
|  | 0002756 |
|  | 0035666 |
|  | 0045087 |
|  | 0009405 |
|  | 0034166 |
|  | 0034134 |
|  | 0034138 |
|  | 0034142 |
|  | 0034146 |
|  | 0034162 |
|  | 0038123 |
|  | 0038124 |
|  | 0002224 |
|  | 0016772 |
|  | 0008063 |
|  | 0034130 |
|  | 0005515 |

---

|  |  |
| --- | --- |
| KEGG ID | hsa:5609 |
|  | mmu:26400 |
|  | rno:363855 |

---

|  |  |
| --- | --- |
| CellType | cells in endometrial stroma |
|  | glandular cells |
|  | trophoblastic cells |
|  | germinal center cells |
|  | squamous epithelial cells |
|  | cells in seminiferus ducts |
|  | non-germinal center cells |
|  | glial cells |
|  | epidermal cells |
|  | Leydig Cells |
|  | respiratory epithelial cells |
|  | neuronal cells |
|  | cells in glomeruli |
|  | myocytes |
|  | follicle cells |
|  | cells in tubules |
|  | ovarian stroma cells |
|  | lymphoid tissue |
|  | exocrine glandular cells |
|  | hematopoietic cells |
|  | urothelial cells |
|  | smooth muscle cells |
|  | islets of Langerhans |
|  | bile duct cells |
|  | cells in red pulp |
|  | Hepatocytes |
|  | decidual cells |
|  | Macrophages |
|  | cells in granular layer |
|  | Pneumocytes |
|  | cells in molecular layer |
|  | Purkinje Cells |
|  | cells in white pulp |

---

|  |  |
| --- | --- |
| Tissue | uterus, pre-menopause |
|  | stomach, lower |
|  | Placenta |
|  | stomach, upper |
|  | Prostate |
|  | lymph node |
|  | vagina |
|  | testis |
|  | rectum |
|  | hippocampus |
|  | vulva/anal skin |
|  | Salivary gland |
|  | nasopharynx |
|  | cerebral cortex |
|  | thyroid gland |
|  | seminal vesicle |
|  | oral mucosa |
|  | Kidney |
|  | cervix, uterine |
|  | Adrenal gland |
|  | tonsil |
|  | skeletal muscle |
|  | Ovary |
|  | Appendix |
|  | Skin |
|  | lateral ventricle |
|  | colon |
|  | small intestine |
|  | Pancreas |
|  | duodenum |
|  | bone marrow |
|  | urinary bladder |
|  | smooth muscle |
|  | Liver |
|  | epididymis |
|  | breast |
|  | spleen |
|  | parathyroid gland |
|  | esophagus |
|  | bronchus |
|  | Lung |
|  | fallopian tube |
|  | Cerebellum |
|  | gall bladder |
|  | heart muscle |
|  | uterus, post-menopause |

---

|  |  |
| --- | --- |
| Organism | Homo sapiens {Organism urn:agi-taxid:9606} |
|  | Mus musculus {Organism urn:agi-taxid:10090} |
|  | Rattus norvegicus {Organism urn:agi-taxid:10116} |
|  | Homo sapiens |
|  | Mus musculus |
|  | Rattus norvegicus |

---

|  |  |
| --- | --- |
| Mouse chromosome position | 8 |
|  | 8 A1.1 |

---

|  |  |
| --- | --- |
| OMIM ID | 603014 |

---

|  |  |
| --- | --- |
| Rat chromosome position | 12p12 |

---

|  |  |
| --- | --- |
| Hugo ID | 6847 |
|  | HGNC:6847 |

---

|  |  |
| --- | --- |
| Human chromosome position | 19p13.2 |
|  | 19p13.3-p13.2 |

---

|  |  |
| --- | --- |
| Swiss-Prot Accession | B4DV95 |
|  | O14733 |
|  | O14733.2 |
|  | Q8CE90 |
|  | Q8CE90.1 |
|  | Q4KSH7 |
|  | D3XAM6 |
|  | Q4KSH7.1 |
|  | B2R9S5 |
|  | D6W659 |
|  | O14648 |
|  | O14816 |
|  | O60452 |
|  | O60453 |
|  | Q1PG43 |
|  | Q8IY10 |
|  | O35406 |
|  | O35720 |
|  | O35871 |
|  | O35872 |
|  | O54780 |
|  | O70242 |
|  | O70243 |
|  | Q8BSP1 |
|  | Q9QWG6 |
|  | Q9R1Z3 |
|  | Q9R1Z4 |
|  | Q9R1Z5 |
|  | Q9R1Z6 |
|  | MP2K7\_HUMAN |
|  | MP2K7\_MOUSE |
|  | MP2K7\_RAT |
|  | B2R9S5\_HUMAN |

---

|  |  |
| --- | --- |
| GenBank ID | NC\_000019 |
|  | XM\_006722800 |
|  | XP\_006722863 |
|  | NM\_001297555 |
|  | NP\_001284484 |
|  | NM\_001297556 |
|  | NP\_001284485 |
|  | NM\_145185 |
|  | NP\_660186 |
|  | NG\_029438 |
|  | AC010336 |
|  | CH471139 |
|  | EAW68962 |
|  | EAW68963 |
|  | EAW68964 |
|  | EAW68965 |
|  | EAW68966 |
|  | EAW68967 |
|  | EAW68968 |
|  | AF003199 |
|  | AAB63374 |
|  | AF006689 |
|  | AAB97813 |
|  | AF013588 |
|  | AAC16272 |
|  | AF013589 |
|  | AAC16273 |
|  | AF014401 |
|  | AAB88048 |
|  | AF022805 |
|  | AAC26142 |
|  | AK098807 |
|  | AK300988 |
|  | BAG62607 |
|  | AK313899 |
|  | BAG36622 |
|  | BC005365 |
|  | BC038295 |
|  | AAH38295 |
|  | BF445461 |
|  | DQ445915 |
|  | ABE03013 |
|  | HY063492 |
|  | HY099294 |
|  | O14733 |
|  | NC\_000074 |
|  | NM\_001164172 |
|  | NP\_001157644 |
|  | NM\_001042557 |
|  | NP\_001036022 |
|  | NM\_011944 |
|  | NP\_036074 |
|  | NM\_001291777 |
|  | NP\_001278706 |
|  | NM\_001291783 |
|  | NP\_001278712 |
|  | NM\_001291778 |
|  | NP\_001278707 |
|  | AC123029 |
|  | CH466566 |
|  | EDL21977 |
|  | EDL21978 |
|  | EDL21979 |
|  | EDL21980 |
|  | EDL21981 |
|  | EDL21982 |
|  | EDL21983 |
|  | U93031 |
|  | AAB63448 |
|  | AB005654 |
|  | BAA24383 |
|  | AF022112 |
|  | AAC16274 |
|  | AF022113 |
|  | AAC16275 |
|  | AF060943 |
|  | AAD15819 |
|  | AF060944 |
|  | AAD15820 |
|  | AF060945 |
|  | AAD15821 |
|  | AF060946 |
|  | AAD15822 |
|  | AF060947 |
|  | AAD15823 |
|  | AK031137 |
|  | BAC27272 |
|  | AK165184 |
|  | BAE38066 |
|  | AK181654 |
|  | AK181910 |
|  | AK196139 |
|  | AK202520 |
|  | AK206802 |
|  | AK211581 |
|  | AK213976 |
|  | AK214564 |
|  | AK218534 |
|  | AK218599 |
|  | AK219049 |
|  | BB842920 |
|  | BC070467 |
|  | AAH70467 |
|  | CO044477 |
|  | U74463 |
|  | AAC53364 |
|  | U74464 |
|  | AAC53365 |
|  | U93030 |
|  | AAB63447 |
|  | Q8CE90 |
|  | NC\_005111 |
|  | XM\_006248769 |
|  | XP\_006248831 |
|  | XM\_008768985 |
|  | XP\_008767207 |
|  | XM\_006248768 |
|  | XP\_006248830 |
|  | NM\_001025425 |
|  | NP\_001020596 |
|  | AC\_000080 |
|  | AABR07034979 |
|  | AAHX01070760 |
|  | CH474084 |
|  | EDL74986 |
|  | EDL74987 |
|  | EDL74988 |
|  | EDL74989 |
|  | EDL74990 |
|  | EDL74991 |
|  | EDL74992 |
|  | AY879265 |
|  | AAX61178 |
|  | GU264001 |
|  | ADC53401 |
|  | Q4KSH7 |
|  | NC\_018930 |
|  | AMYH02035411 |
|  | DQ891274 |
|  | ABM82200 |
|  | DQ894462 |
|  | ABM85388 |
|  | AC\_000030 |
|  | AAHY01067537 |
|  | XM\_005272489 |
|  | XP\_005272546 |
|  | XM\_005272490 |
|  | XP\_005272547 |
|  | AC\_000151 |
|  | ABBA01010877 |
|  | ABBA01010878 |
|  | XM\_006508805 |
|  | XP\_006508868 |
|  | AK049267 |
|  | NT\_077812 |
|  | NW\_001838480 |
|  | NW\_927173 |
|  | AC\_000062 |
|  | CR604614 |
|  | NT\_039455 |
|  | NW\_001030882 |
|  | AF026216 |
|  | AAB81848 |
|  | AK028772 |
|  | BAC26111 |
|  | NW\_001084669 |
|  | NW\_047365 |
|  | AABR06069894 |

---

|  |  |
| --- | --- |
| Swiss-Prot ID | MP2K7\_HUMAN |
|  | MP2K7\_MOUSE |
|  | MP2K7\_RAT |
|  | B2R9S5\_HUMAN |

---

|  |  |
| --- | --- |
| Cell Localization | Nucleus |
|  | Cytoplasm |

---

|  |  |
| --- | --- |
| Ensembl ID | ENSG00000076984 |
|  | ENSP00000381070.2 |
|  | ENST00000397983.7 |
|  | ENSP00000381068.3 |
|  | ENST00000397981.7 |
|  | ENSP00000381066.3 |
|  | ENST00000397979.3 |
|  | ENSMUSG00000002948 |
|  | ENSMUSP00000003027.7 |
|  | ENSMUST00000003027.13 |
|  | ENSMUSP00000054512.4 |
|  | ENSMUST00000062686.10 |
|  | ENSMUSP00000106627.1 |
|  | ENSMUST00000110999.7 |
|  | ENSMUSP00000106623.1 |
|  | ENSMUST00000110995.7 |
|  | ENSMUSP00000106622.1 |
|  | ENSMUST00000110994.8 |
|  | ENSMUSP00000106626.2 |
|  | ENSMUST00000110998.8 |
|  | ENSRNOG00000001047 |
|  | ENSRNOP00000058535.3 |
|  | ENSRNOT00000061821.3 |
|  | ENSRNOP00000072160.1 |
|  | ENSRNOT00000084460.1 |
|  | ENSP00000381070 |
|  | ENST00000397983 |
|  | ENSP00000381068 |
|  | ENST00000397981 |
|  | ENSP00000381066 |
|  | ENST00000397979 |
|  | ENSMUSG00000109061 |
|  | ENSMUSP00000003027 |
|  | ENSMUST00000003027 |
|  | ENSMUSP00000054512 |
|  | ENSMUST00000062686 |
|  | ENSMUSP00000106627 |
|  | ENSMUST00000110999 |
|  | ENSMUSP00000106623 |
|  | ENSMUST00000110995 |
|  | ENSMUSP00000106622 |
|  | ENSMUST00000110994 |
|  | ENSMUSP00000106626 |
|  | ENSMUST00000110998 |
|  | ENSRNOP00000058535 |
|  | ENSRNOT00000061821 |
|  | ENSRNOP00000072160 |
|  | ENSRNOT00000084460 |

---

|  |  |
| --- | --- |
| MGI ID | MGI:1346871 |
|  | 1346871 |

---

|  |  |
| --- | --- |
| RGD ID | 1560043 |

---

|  |  |
| --- | --- |
| Unigene ID | Hs.531754 |
|  | Mm.3906 |
|  | Rn.162081 |

---

|  |  |
| --- | --- |
| Homologene ID | 56548 |

---

|  |  |
| --- | --- |
| Shape | Sickle-vertex |

---

|  |  |
| --- | --- |
| IPI ID | IPI00023636 |
|  | IPI00302112 |
|  | IPI00745806 |
|  | IPI00794495 |
|  | IPI00779757 |
|  | IPI00117840 |
|  | IPI00228989 |
|  | IPI00280475 |
|  | IPI00749972 |
|  | IPI00816840 |
|  | IPI00816864 |
|  | IPI00816890 |
|  | IPI00816911 |
|  | IPI00747921 |
|  | IPI00641984 |
|  | IPI00639798 |
|  | IPI00189128 |

---

|  |  |
| --- | --- |
| Ensembl Transcript ID | ENST00000397979 |

---

|  |  |
| --- | --- |
| ENSEMBL | ENSG00000076984 |

---

|  |  |
| --- | --- |
| ProteinAtlas SubcellularLocation | Main location: ; Other location: ; Expression type: Staining; Reliability: Uncertain |

---

|  |  |
| --- | --- |
| Ensembl Gene ID | ENSG00000076984 |

---

|  |  |
| --- | --- |
| ProteinAtlas NormalTissue | Tissue: vulva/anal skin; Cell type: epidermal cells; Level: Moderate; Expression type: Staining; Reliability: Supportive |
|  | Tissue: pancreas; Cell type: islets of Langerhans; Level: Weak; Expression type: Staining; Reliability: Supportive |
|  | Tissue: pancreas; Cell type: exocrine glandular cells; Level: Moderate; Expression type: Staining; Reliability: Supportive |
|  | Tissue: spleen; Cell type: cells in red pulp; Level: Weak; Expression type: Staining; Reliability: Supportive |
|  | Tissue: smooth muscle; Cell type: smooth muscle cells; Level: Weak; Expression type: Staining; Reliability: Supportive |
|  | Tissue: adrenal gland; Cell type: glandular cells; Level: Low; Expression type: APE; Reliability: High |
|  | Tissue: appendix; Cell type: glandular cells; Level: Medium; Expression type: APE; Reliability: High |
|  | Tissue: appendix; Cell type: lymphoid tissue; Level: Medium; Expression type: APE; Reliability: High |
|  | Tissue: bone marrow; Cell type: hematopoietic cells; Level: High; Expression type: APE; Reliability: High |
|  | Tissue: breast; Cell type: glandular cells; Level: Medium; Expression type: APE; Reliability: High |
|  | Tissue: bronchus; Cell type: respiratory epithelial cells; Level: Medium; Expression type: APE; Reliability: High |
|  | Tissue: cerebellum; Cell type: cells in granular layer; Level: Low; Expression type: APE; Reliability: High |
|  | Tissue: cerebellum; Cell type: cells in molecular layer; Level: Low; Expression type: APE; Reliability: High |
|  | Tissue: cerebellum; Cell type: Purkinje cells; Level: Low; Expression type: APE; Reliability: High |
|  | Tissue: cerebral cortex; Cell type: glial cells; Level: Medium; Expression type: APE; Reliability: High |
|  | Tissue: cerebral cortex; Cell type: neuronal cells; Level: Medium; Expression type: APE; Reliability: High |
|  | Tissue: cervix, uterine; Cell type: glandular cells; Level: Medium; Expression type: APE; Reliability: High |
|  | Tissue: cervix, uterine; Cell type: squamous epithelial cells; Level: Low; Expression type: APE; Reliability: High |
|  | Tissue: colon; Cell type: glandular cells; Level: Medium; Expression type: APE; Reliability: High |
|  | Tissue: duodenum; Cell type: glandular cells; Level: Medium; Expression type: APE; Reliability: High |
|  | Tissue: epididymis; Cell type: glandular cells; Level: Low; Expression type: APE; Reliability: High |
|  | Tissue: esophagus; Cell type: squamous epithelial cells; Level: Medium; Expression type: APE; Reliability: High |
|  | Tissue: fallopian tube; Cell type: glandular cells; Level: Medium; Expression type: APE; Reliability: High |
|  | Tissue: gall bladder; Cell type: glandular cells; Level: Medium; Expression type: APE; Reliability: High |
|  | Tissue: heart muscle; Cell type: myocytes; Level: None; Expression type: APE; Reliability: High |
|  | Tissue: hippocampus; Cell type: glial cells; Level: Low; Expression type: APE; Reliability: High |
|  | Tissue: hippocampus; Cell type: neuronal cells; Level: Low; Expression type: APE; Reliability: High |
|  | Tissue: kidney; Cell type: cells in glomeruli; Level: Medium; Expression type: APE; Reliability: High |
|  | Tissue: kidney; Cell type: cells in tubules; Level: Medium; Expression type: APE; Reliability: High |
|  | Tissue: lateral ventricle; Cell type: glial cells; Level: Low; Expression type: APE; Reliability: High |
|  | Tissue: lateral ventricle; Cell type: neuronal cells; Level: Low; Expression type: APE; Reliability: High |
|  | Tissue: liver; Cell type: bile duct cells; Level: Low; Expression type: APE; Reliability: High |
|  | Tissue: liver; Cell type: hepatocytes; Level: Low; Expression type: APE; Reliability: High |
|  | Tissue: lung; Cell type: macrophages; Level: Medium; Expression type: APE; Reliability: High |
|  | Tissue: lung; Cell type: pneumocytes; Level: None; Expression type: APE; Reliability: High |
|  | Tissue: lymph node; Cell type: germinal center cells; Level: Medium; Expression type: APE; Reliability: High |
|  | Tissue: lymph node; Cell type: non-germinal center cells; Level: Medium; Expression type: APE; Reliability: High |
|  | Tissue: nasopharynx; Cell type: respiratory epithelial cells; Level: Medium; Expression type: APE; Reliability: High |
|  | Tissue: oral mucosa; Cell type: squamous epithelial cells; Level: Low; Expression type: APE; Reliability: High |
|  | Tissue: ovary; Cell type: follicle cells; Level: Low; Expression type: APE; Reliability: High |
|  | Tissue: ovary; Cell type: ovarian stroma cells; Level: Low; Expression type: APE; Reliability: High |
|  | Tissue: pancreas; Cell type: exocrine glandular cells; Level: Medium; Expression type: APE; Reliability: High |
|  | Tissue: pancreas; Cell type: islets of Langerhans; Level: Low; Expression type: APE; Reliability: High |
|  | Tissue: parathyroid gland; Cell type: glandular cells; Level: Medium; Expression type: APE; Reliability: High |
|  | Tissue: placenta; Cell type: decidual cells; Level: Low; Expression type: APE; Reliability: High |
|  | Tissue: placenta; Cell type: trophoblastic cells; Level: Medium; Expression type: APE; Reliability: High |
|  | Tissue: prostate; Cell type: glandular cells; Level: Medium; Expression type: APE; Reliability: High |
|  | Tissue: rectum; Cell type: glandular cells; Level: Medium; Expression type: APE; Reliability: High |
|  | Tissue: salivary gland; Cell type: glandular cells; Level: Low; Expression type: APE; Reliability: High |
|  | Tissue: seminal vesicle; Cell type: glandular cells; Level: Medium; Expression type: APE; Reliability: High |
|  | Tissue: skeletal muscle; Cell type: myocytes; Level: None; Expression type: APE; Reliability: High |
|  | Tissue: skin; Cell type: epidermal cells; Level: Low; Expression type: APE; Reliability: High |
|  | Tissue: small intestine; Cell type: glandular cells; Level: Medium; Expression type: APE; Reliability: High |
|  | Tissue: smooth muscle; Cell type: smooth muscle cells; Level: Low; Expression type: APE; Reliability: High |
|  | Tissue: spleen; Cell type: cells in red pulp; Level: Low; Expression type: APE; Reliability: High |
|  | Tissue: spleen; Cell type: cells in white pulp; Level: Low; Expression type: APE; Reliability: High |
|  | Tissue: stomach, lower; Cell type: glandular cells; Level: Medium; Expression type: APE; Reliability: High |
|  | Tissue: stomach, upper; Cell type: glandular cells; Level: Medium; Expression type: APE; Reliability: High |
|  | Tissue: testis; Cell type: cells in seminiferus ducts; Level: High; Expression type: APE; Reliability: High |
|  | Tissue: testis; Cell type: Leydig cells; Level: Low; Expression type: APE; Reliability: High |
|  | Tissue: thyroid gland; Cell type: glandular cells; Level: Low; Expression type: APE; Reliability: High |
|  | Tissue: tonsil; Cell type: germinal center cells; Level: Medium; Expression type: APE; Reliability: High |
|  | Tissue: tonsil; Cell type: non-germinal center cells; Level: Medium; Expression type: APE; Reliability: High |
|  | Tissue: tonsil; Cell type: squamous epithelial cells; Level: Low; Expression type: APE; Reliability: High |
|  | Tissue: urinary bladder; Cell type: urothelial cells; Level: Medium; Expression type: APE; Reliability: High |
|  | Tissue: uterus, post-menopause; Cell type: cells in endometrial stroma; Level: None; Expression type: APE; Reliability: High |
|  | Tissue: uterus, post-menopause; Cell type: glandular cells; Level: Low; Expression type: APE; Reliability: High |
|  | Tissue: uterus, pre-menopause; Cell type: cells in endometrial stroma; Level: Low; Expression type: APE; Reliability: High |
|  | Tissue: uterus, pre-menopause; Cell type: glandular cells; Level: Medium; Expression type: APE; Reliability: High |
|  | Tissue: vagina; Cell type: squamous epithelial cells; Level: Medium; Expression type: APE; Reliability: High |
|  | Tissue: vulva/anal skin; Cell type: epidermal cells; Level: Medium; Expression type: APE; Reliability: High |

---
